# The Intelligence of Dual Simplex Method to Solve Linear Fractional Fuzzy Transportation Problem

**DOI:** 10.1155/2015/103618

**Published:** 2015-02-24

**Authors:** S. Narayanamoorthy, S. Kalyani

**Affiliations:** Department of Applied Mathematics, Bharathiar University, Coimbatore, Tamil Nadu 641 046, India

## Abstract

An approach is presented to solve a fuzzy transportation problem with linear fractional fuzzy objective function. In this proposed approach the fractional fuzzy transportation problem is decomposed into two linear fuzzy transportation problems. The optimal solution of the two linear fuzzy transportations is solved by dual simplex method and the optimal solution of the fractional fuzzy transportation problem is obtained. The proposed method is explained in detail with an example.

## 1. Introduction

The transportation problem refers to a special case of linear programming problem. The basic transportation problem was developed by Hitchcock in 1941 [1]. In mathematics and economics transportation theory is a name given to the study of optimal transportation and allocation of resources. The problem was formalized by the French Mathematician Gaspard Monge. Tolstoi was one of the first person to study the transportation problem mathematically. Transportation problem deals with the distribution of single commodity from various sources of supply to various destinations of demand in such a manner that the total transportation cost is minimized. In order to solve a transportation problem the decision parameters such as availability, requirements and the unit transportation cost of the model must be fixed at crisp values.

When applying OR-methods to engineering problems, for instance, the problems to be modelled and solved are normally quite clear cut, well described, and crisp. They can generally be modelled and solved by using classical mathematics which is dichotomous in character. If vagueness enters, it is normally of the stochastic kind which can properly be modelled by using probability theory. This is true for many areas such as inventory theory, traffic control, and scheduling, in which probability theory is applied via queuing theory. One of the most important areas of the kind in which the vagueness is of a different kind compared to randomness is probably that of decision making. In decision making the human factor enters with all its vagueness of perception, subjectivity, and attitudes of goals and of conceptions. In transportation problem supply, demand and unit transportation cost may be uncertain due to several factors; these imprecise data may be represented by fuzzy numbers. When the demand, supply, or the unit cost of the transportation problem takes a fuzzy value then the optimum value of that problem is also fuzzy, so it becomes a fuzzy transportation problem.

Linear fractional programming deals with that class of mathematical programming problem in which the relations among the variables are linear; the constraint relation must be in linear form and the objective function to be optimized must be a ratio of two linear functions. The field of linear fractional programming problem was developed by Hungarian mathematician B. Matros in 1960. The linear fractional programming problem is an important planning tool for the past decades which is applied to different disciplines like engineering, business, finance, and economics. Linear fractional problem is generally used for modelling real life problems with one or more objective(s) such as profit-cost, inventory-sales, actual cost-standard cost, and output-employee; multiple level programming problems are frequently encountered in a hierarchical organization, manufacturing plant, logistic companies, and so forth. From practical point of view, it becomes necessary to consider the large structured programming problem. Even it is theoretically possible to solve this problem, in practical it is not easy to solve. There are certain limitations which restrict the endeavours of the analyst. Chief among these limitations is the problem of dimensionality. This suggests the idea of developing methods of solution that should not use simultaneously all the data of the problem; one such approach is the decomposition principle due to Dantzig [2] for linear programs. The principle requires the solution of a series of linear programming problems of smaller size than the original problem. In this paper, we proposed a new method to find the optimal solution of the fractional fuzzy transportation problem based on dual simplex approach.

This paper is organised as follows. In Section 2 related work is presented.  Section 3 provides preliminary background on fuzzy set theory. The linear fractional fuzzy programming problem is explained in Section 4. In Section 5 problem formulation of fractional fuzzy transportation problem appeared. The procedure for the proposed method is given in Section 6. An illustrative example is provided to explain this proposed method and the conclusions are given in Sections 7 and 8, respectively.

## 2. Related Work

Fractional fuzzy transportation problem is a special type of linear programming problem and it is an active area of research. There are a lot of articles in this area which cannot be reviewed completely and only a few of them are reviewed here. Charnes and Cooper [3] developed a transformation technique *z* = 1/(*q*′*x* + *β*) to convert fractional programming method to linear programming method, Swarup [4] introduced simplex type algorithm for fractional program, and Bitran and Novaes [5] converted linear fractional problem into a sequence of linear programs and the method is widely accepted. Tantawy [6] developed a dual solution technique for the fractional program. Hasan and Acharjee [7] used the conversion of linear fractional problem into single linear programming and computed in* MATHEMATICA*. Joshi and Gupta [8] solved the linear fractional problem by primal-dual approach. Sharma and Bansal [9] proposed a method based on simplex method in which the variables are extended. Jain et al. [10] proposed ABC algorithm for solving linear fractional programming problem using C-language. Sulaiman and Basiya [11] used transformation technique.

Verma et al. [12] developed an algorithm which ranks the feasible solutions of an integer fractional programming problem in decreasing order of the objective function values. Metev and Yordanova-Markova [13] applied reference point method. Pandian and Jayalakshmi [14] used decomposition method and the denominator restriction method to convert fractional problem to linear problem. Chandra [15] proposed decomposition principle for solving linear fractional problem. Sharma and Bansal [9] gave an integer solution for linear fractional problem. Dheyah [16] gave optimal solution for linear fractional problem with fuzzy numbers. Güzel et al. [17] proposed an algorithm using first order Taylor series to convert interval linear fractional transportation problem into linear multiobjective problem. Madhuri [18] gave a solution procedure to minimum time for linear fractional transportation problem with impurities. Jain and Arya [19] solved an inverse transportation problem with linear fractional objective function.

Motivated by the work of Güzel et al. [17], in this paper the author proposes a new method to find the optimal solution of the fractional fuzzy transportation problem. Some of the limitations in the existing methods are that, in Charnes and Coopers methods to obtain the solution, one needs to solve the linear problem by two-phase method in each step which becomes lengthy and clumsy. In Bitran and Novae's method one needs to solve a sequence of problems which sometimes may need many iterations. In this method if *p*(*x*) < 0 for all *x* then this method fails. In Swarup's method when the constraints are not in canonical form then this method becomes lengthy and its computational process is complicated in each iteration. In Harvey's method he converts the linear fractional problem max⁡⁡*z* = (*cx* + *α*)/(*dx* + *β*) to linear problem under the assumption that *β* ≠ 0. If *β* = 0 this method fails.

To overcome these limitations, in this paper a new method is proposed such that the linear fractional transportation problem is decomposed into two linear fuzzy transportation problems under the assumption that *α*, *β* values are zero. These linear transportation problems are solved by dual simplex method.

## 3. Preliminaries

In this section, we present the most basic notations and definitions, which are used throughout this work. We start with defining a fuzzy set.

### 3.1. Fuzzy Set [20]

Let *X* be a set. A fuzzy set *A* on *X* is defined to be a function *A* : *X* → [0, 1] or *μ*_*A*_ : *X* → [0, 1]. Equivalently, a fuzzy set is defined to be the class of objects having the following representation: *A* = {(*x*, *μ*_*A*(*x*)_), *x* ∈ *X*} where *μ*_*A*_ : *X* → [0, 1] is a function called membership function of *A*.

### 3.2. Fuzzy Number [20]

The fuzzy number *A* is a fuzzy set whose membership function satisfies the following conditions.

- *μ*_*A*(*x*)_ is piecewise and continuous.
- A fuzzy set *A* of the universe of discourse *X* is convex.
- A fuzzy set of the universe of discourse *X* is called a normal fuzzy set if there exists *x*_*i*_ ∈ *X*.

### 3.3. Trapezoidal Fuzzy Number [20]

A fuzzy number $\tilde{a}=(a_{1},a_{2},a_{3},a_{4})$ is said to be a trapezoidal fuzzy number if its membership function is given by

(1) $$\begin{array}{c} \mu_{\tilde{a}}=\left\{\begin{array}{cc} 0, & x\;\leq \;a_{1}\\ \frac{x-a}{b-a}, & a_{1}\leq x\leq a_{2}\\ 1, & {\;a}_{2}\;\leq x\;\leq \;a_{3}\\ \frac{c-x}{d-c}, & a_{3}\leq x\;\leq \;a_{4}\\ 0, & x\geq \;a_{4}. \end{array}\right. \end{array}$$

### 3.4. Arithmetic Operations

Let $\tilde{a}=\left(a_{1},a_{2},a_{3},a_{4}\right)$ and $\tilde{b}=(b_{1},b_{2},b_{3},b_{4})$ be two trapezoidal fuzzy numbers; the arithmetic operators on these numbers are as follows.

Addition:

(2) $$\begin{array}{c} \tilde{a}+\tilde{b}=\left(a_{1}+b_{1},a_{2}+b_{2},a_{3}+b_{3},a_{4}+b_{4}\right). \end{array}$$

Subtraction:

(3) $$\begin{array}{c} \tilde{a}-\tilde{b}=\left(a_{1}-b_{1},a_{2}-b_{2},a_{3}-b_{3},a_{4}-b_{4}\right). \end{array}$$

Scalar multiplication:

(4) $$\begin{array}{c} k\tilde{a}=\left({ka}_{1},{ka}_{2},{ka}_{3},{ka}_{4}\right),\;k>0,\\ k\tilde{a}=\left({ka}_{4},{ka}_{3},{ka}_{2},{ka}_{1}\right),\;k<0. \end{array}$$

### 3.5. Ranking Function

The following ranking function defined here is used to compare the fuzzy values when we perform the dual simplex method. A ranking function *R* : *F*(*R*) → *R*, which maps each fuzzy number into the real line. *F*(*R*) represents the set of all trapezoidal fuzzy numbers. If *R* is any ranking function, then $R\left(\tilde{a}\right)=\left({\tilde{a}}_{1}+{\tilde{a}}_{2}+{\tilde{a}}_{3}+{\tilde{a}}_{4}\right)/4$.

### 3.6. Efficient Point

A feasible point *x* ^0^ ∈ *P* is said to be an efficient solution for *P* if there exists no other feasible point *x* of the problem *P* such that *z*_*i*_(*x*) ≥ *z*_*i*_(*x* ^0^) and *i* = 1, 2.

### 3.7. Ideal Solution

Let *x* ^0^ be the optimal solution to the problem *P*_*t*_, *t* = 1, 2; then the value of the objective function *z*_*t*_(*x* ^0^), *t* = 1, 2, is called the ideal solution to the problem *P*.

### 3.8. Best Compromise Solution

An efficient solution *x* ^0^ ∈ *P* is said to be the best compromise solution to the problem *P*, if the distance between the ideal solution and the objective value at *x* ^0^, *z*_*t*_(*x* ^0^), *t* = 1, 2, is minimum among the efficient solution to the problem *P*.

**Lemma 1 .**

The feasible solution for the problem *P* is considered to be efficient if and only if there exist no other feasible solutions for which we obtain a better value at least for one criterion for which the value of the rest of criteria remains unmodified.

**Theorem 2 .**

If *X*_*t*_ is the set of efficient points for problem *P*_*t*_, *t* = 1, 2, then *X*_*t*_ is a subset of the set of all efficient points *X* of *P*.

**Proof**

Let *X*_1_ be the set of efficient points of *P*_1_. Let *X*_2_ be the set of efficient points of *P*_2_. As *X*_*t*_ ⊂ *X*, *t* = 1, 2. The set of all efficient points of *P*_1_, *P*_2_ is contained in *P*.

**Theorem 3 .**

Let ${\overset{-}{x}}^{0}=\{x_{ij},x_{ij}\in P\}$ be an optimal solution of *P*_1_ and let ${\underline{x}}^{0}=\{x_{ij},x_{ij}\in P_{2}\}$ be an optimal solution of *P*_2_; then $x={\overset{-}{x}}^{0}/{\underline{x}}^{0}$ is an optimal solution of the given problem *P* where all of *x*_11_, *x*_12_, *x*_21_, *x*_22_,… are elements of *P*_1_, *P*_2_.

**Proof**

Let ${\overset{-}{x}}^{0}$ be an optimal solution of *P*_1_. This solution is an efficient solution if there is no feasible solution; these sets of efficient solutions are contained in *x*. Similarly the set of efficient solutions of *P*_2_ are contained in *x*. So, by Theorem 2, these solutions are the set of efficient solutions of *P*.

## 4. Introduction to Linear Fractional Fuzzy Programming Problem (LFFPP)

One of the main aims of this paper is to extend the linear fractional problem into linear fractional fuzzy programming problem.

The LFFPP can be presented in the following way:

(5) max⁡ hhhhfx=p(x)q(x)subject  to x∈S⊂Rn,

where *p*(*x*) and *q*(*x*) are linear functions and the set *S* is defined as *S* = {*x*/*Ax* = *b*, *x* > 0}. Here *A* is a fuzzy *m* × *n* matrix; we will suppose that *S* is a bounded polyhedron and *q*(*x*) > 0 for all *x* ∈ *S*. It is well known that the goal function in max *f*(*x*) has a global maximum function *S* and has not any other local maxima. This maximum is obtained at an extreme point of *S*.

LFFPP (5) can now be written as

(6) max⁡ hhhlhp(x)subject  tohhx∈S⊂Rnmax⁡ hhhhq(x)subject  to x∈S⊂Rn.

Then by Theorem 3, if *p*(*x*)/*q*(*x*) > 0  ∀*i* and ∀*x* ∈ *S* then the set of efficient points for problem *P*_*t*_, *t* = 1, 2, is a subset of the set of all efficient points of *P*.

## 5. Problem Formulation 

The proposed fuzzy transportation model is based on the following assumption.

*Index Set.* Consider the following

 
*i* is source index for all *i* = 1,2, 3,…, *m*.
 
*j* is destination index for all *j* = 1,2, 3,…, *n*.

*Parameters.* Consider the following

 
*x*_*ij*_ is the number of units transported from source *i* to destination *j*.
 
*c*_*ij*_ is per unit profit cost of transportation from source *i* to destination *j*.
 
*d*_*ij*_ is per unit cost of transportation from source *i* to destination *j*.

*Objective Function.* Consider the following.

(i) Fractional transportation problem
  (7) $$\begin{array}{c} \min z=\frac{\sum_{i=1}^{m}\sum_{j=1}^{n}c_{ij}x_{ij}}{\sum_{i=1}^{m}\sum_{j=1}^{n}d_{ij}x_{ij}}. \end{array}$$
  The values of *c*_*ij*_ and *d*_*ij*_ are considered to be trapezoidal fuzzy number.

*Constraints.* Consider the following.

(i) Constraints on supply available for all sources *i*

(8) $$\begin{array}{c} \overset{m}{\underset{i=1}{\sum }}x_{ij}=a_{i}\;\forall i. \end{array}$$

(ii) Constraints on demand for each destination *j*

(9) $$\begin{array}{c} \overset{n}{\underset{j=1}{\sum }}x_{ij}=b_{j}\;\forall j. \end{array}$$

*Nonnegative Constraints.* Consider the following.

(i) Nonnegative constraints on decision variables

(10) $$\begin{array}{c} x_{ij}\geq 0. \end{array}$$

The above LFFTP will have a basic feasible solution only when ∑_*i*=1_^*m*^ *a*_*i*_ = ∑_*j*=1_^*n*^ *b*_*j*_.

## 6. Procedure for Proposed Method

Here we present a new method for solving linear fractional fuzzy transportation problem.

Consider the linear fractional fuzzy transportation problem as

(11) P: min⁡hhhhhhz=∑i=1m  ∑j=1ncijxij∑i=1m∑j=1ndijxijP: subject  to ∑i=1mxij=aiP: subject  o ∑j=1nxij=bjP: subject  o xij≥0.

Since the objective function is a fractional objective function, we take the problem *P* into two linear fuzzy transportation problems by (6) as follows:

(12) P1:lmin⁡P1:h zN=∑i=1m∑j=1ncijxijNP1: subject  to ∑i=1mxijN=aiP1: subjectlo ∑j=1nxijN=bjP1: subjectolt xijN≥0P1:lmin⁡P1:hl zD=∑i=1m∑j=1ncijxijDP1: subject  to ∑i=1mxijD=aiP1: subject  t ∑j=1nxijD=bjP1: subject  tl xijD≥0.

We solve these two linear fuzzy transportation problems by dual simplex methods. Thus the solution is obtained for min⁡*z* ^*N*^ and min⁡*z* ^*D*^ separately. By Theorem 3, the optimum solution of *P* is obtained when the optimal solution of *P*_1_ and *P*_2_ is obtained.

## 7. Numerical Example

To illustrate the proposed method, consider the following example for linear fractional fuzzy transportation problem:

(13) P: min⁡hhhhhhz=0,2,4,6x11+1,2,6,7x12P: min⁡  hhhhhh+1,4,5,6x21+3,4,5,8x22P: min⁡  hhhhhh·0,1,3,4x11+2,3,5,6x12P: min⁡  hhhhhhhhl+1,3,5,7x21+2,6,7,9x22−1P: subject  to x11+x12≤60P: subject  to x21+x22≤45P: subject  to x11+x21≥50P: subject  to x12+x22≥55P: subject  to x11,x12,x21,x22≥0.

The problem *P* is a linear fractional fuzzy transportation problem in which the objective function has trapezoidal fuzzy number; supply and demand values are crisp values. Now by our computation technique the above problem can be written as

(14) P1: min⁡hhhhhhzN=0,2,4,6x11+1,2,6,7x12P1: minhhhhhhhhhh+1,4,5,6x21+(3,4,5,8)x22P1: subject  to x11+x12≤60P1: minhhhhhhx21+x22≤45P1: minhhhhhhx11+x21≥50P1: minhhhhhhx12+x22≥55P1: minhhhhhhx11,x12,x21,x22≥0,P2: min⁡hhhhhhzD=0,1,3,4x11+2,3,5,6x12hhhhhhhhhhhhhhhhh+1,3,5,7x21+2,6,7,9x22P2: subject  to x11+x12≤60P2: subject  to x21+x22≤45P2: subject  to x11+x21≥50P2: subject  to x12+x22≥55P2: subject  to x11,x12,x21,x22≥0.

Consider *P*_1_: The linear problem can be expressed in standard form as

(15) P1: min⁡hhhhhlhzN=0,2,4,6x11+1,2,6,7x12hhhhhhhhhhhhhhhhhh+1,4,5,6x21+(3,4,5,8)x22P1: subject  to x11+x12+s1=60P1:subject  toh x21+x22+s2=45P1:subject  toh x11+x21−s3=50P1:subject  toh x12+x22−s4=55P1:subject  toh x11,x12,x21,x22,s1,s2,s3,s4≥0.

Now *P*_1_ is solved by the dual simplex method.

The initial iteration of the problem is given in Table 1.

From the initial iteration we can see that the nonbasic variable *X*_12_ enters the basis and the basic variable *S*_4_ leaves the basis.

Proceeding the dual simplex method and after few iterations we get Table 2. In Table 2 all the values of *X*_*B*_ are positive and the optimum solution is obtained as follows:

(16) min⁡⁡zN=100,390,755,995,x11=5, x12=55, x21=45.

Consider *P*_2_: The linear problem can be expressed in standard form as

(17) P2: min⁡hhhhhhzD=0,1,3,4x11+2,3,5,6x12hhhhhhhhhhhhhhhhhh+1,3,5,7x21+2,6,7,9x22P2: subject  to x11+x12+S1=60P2: subject  to x21+x22+S2=45P2: subject  to x11+x21−S3=50P2: subject  to x12+x22−S4=55P2: subject  to x11,x12,x21,x22,S1,S2,S3,S4≥0.

Now *P*_2_ is solved by the dual simplex method.

The initial iteration of the problem is given in Table 3.

From the initial iteration we can see that the nonbasic variable *X*_12_ enters the basis and the basic variable *S*_4_ leaves the basis.

Proceeding the dual simplex method and after few iterations we get Table 4. In Table 4 all the values of *X*_*B*_ are positive and the optimum solution is obtained as follows:

(18) min⁡⁡zD=155,305,515,665,x11=5, x12=55, x21=45.

By Theorem 3 the optimum solution of the fractional fuzzy transportation problem is obtained.

Thus

(19) $$\begin{array}{c} \min z=\frac{\left(100,390,755,995\right)}{\left(155,305,515,665\right)}. \end{array}$$

## 8. Conclusion

The present paper proposes a method for solving linear fractional fuzzy transportation problem where the trapezoidal fuzzy numbers are used in the objective function. The limitations of the existing methods are discussed. The advantage of the computational technique is that the method works even when *α*, *β* values are zero. The dual simplex method is one of the best methods to get the optimal solution. The method can be easily implemented to solve any type of transportation problem. The solution procedures have been illustrated by an example. The result shows the high efficiency of the solution and this can be extended to fractional quadratic problems. The simplex method can be used instead of dual simplex if all the constraints are of ≤ type constraints.

## Figures and Tables

**Table 1:** 

		*C*_*j*_	(−6, −4, −2, 0)	(−7, −6, −2, −1)	(−6, −5, −4, −1)	(−8, −5, −4, −3)	0	0	0	0
	*Y*_*B*_	*X*_*B*_	*X*_11_	*X*_12_	*X*_21_	*X*_22_	*S*_1_	*S*_2_	*S*_3_	*S*_4_
0	*S*_1_	60	1	1	0	0	1	0	0	0
0	*S*_2_	45	0	0	1	1	0	1	0	0
0	*S*_3_	−50	−1	0	−1	0	0	0	1	0
0	*S*_4_	−55	0	−1	0	−1	0	0	0	1
*z* = 0			(0, 2, 4, 6)	(1, 2, 6, 7)	(1, 4, 5, 6)	(3, 4, 5, 8)	0	0	0	0
*R*(⋯)			3	4	4	5	0	0	0	0
				↑						↓

**Table 2:** 

		*C*_*j*_	(−6, −4, −2, 0)	(−7, −6, −2, −1)	(−6, −5, −4, −1)	(−8, −5, −4, −3)	0	0	0	0
	*Y*_*B*_	*X*_*B*_	*X*_11_	*X*_12_	*X*_21_	*X*_22_	*S*_1_	*S*_2_	*S*_3_	*S*_4_
(−8, −5, −4, −3)	*X*_21_	45	0	0	1	1	−1	0	−1	−1
0	*S*_2_	0	0	0	0	0	1	1	1	1
(−6, −5, −4, −1)	*X*_11_	5	1	0	0	−1	1	0	0	1
(−7, −6, −2, −1)	*X*_12_	55	0	1	0	1	0	0	0	−1
*z* = (−995, − 755, − 390, − 100)			0	0	0	(−5, 8, 6, 9)	(−5, 0, 3, 6)	0	(1, 4, 5, 6)	(−4, 2, 9, 13)

**Table 3:** 

		*C*_*j*_	(−4, −3, −1, 0)	(−6, −5, −3, −2)	(−7, −5, −3, −1)	(−9, −7, −6, −2)	0	0	0	0
	*Y*_*B*_	*X*_*B*_	*X*_11_	*X*_12_	*X*_21_	*X*_22_	*S*_1_	*S*_2_	*S*_3_	*S*_4_
0	*S*_1_	60	1	1	0	0	1	0	0	0
0	*S*_2_	45	0	0	1	1	0	1	0	0
0	*S*_3_	−50	−1	0	−1	0	0	0	1	0
0	*S*_4_	−55	0	−1	0	−1	0	0	0	1
*z* = 0			(0, 1, 3, 4)	(2, 3, 5, 6)	(1, 3, 5, 7)	(2, 6, 7, 9)	0	0	0	0
*R*(⋯)			2	4	4	6	0	0	0	0
				↑						↓

**Table 4:** 

		*C*_*j*_	(−4, −3, −1, 0)	(−6, −5, −3, −2)	(−7, −5, −3, −1)	(−9, −7, −6, −2)	0	0	0	0
	*Y*_*B*_	*X*_*B*_	*X*_11_	*X*_12_	*X*_21_	*X*_22_	*S*_1_	*S*_2_	*S*_3_	*S*_4_
(−7, −5, −3, −1)	*X*_21_	45	0	0	1	1	−1	0	−1	−1
0	*S*_2_	0	0	0	0	0	1	1	1	1
(−4, −3, −1, 0)	*X*_11_	5	1	0	0	−1	1	0	0	1
(−6, −5, −3, −2)	*X*_12_	55	0	1	0	1	0	0	0	−1
*z* = (−665, −515, −305, −155)			0	0	0	(8, −2, 3, 3)	(−3, 0, 4, 7)	0	(1, 3, 5, 7)	(−1, 3.9, 13)
